# Liver ultrasound: Normal anatomy and pathologic findings

**DOI:** 10.1016/j.sopen.2024.05.002

**Published:** 2024-05-10

**Authors:** Natasha Leigh, Chet W. Hammill

**Affiliations:** Department of Surgery, Division of Hepatopancreaticobiliary Surgery, Washington University, St Louis, MO 63110, USA

**Keywords:** Liver, Ultrasound

## Abstract

The goal of this article is to give an overview of intraoperative liver ultrasound, including the indications, different ultrasound techniques, and the ultrasound appearance of normal anatomy, more common anatomic variations, and common hepatic tumors.

## Introduction

Intraoperative ultrasound of the liver has become an essential tool in liver surgery, used for confirmation of the anatomy and pathology at the beginning of the case and surgical guidance throughout the case. The routine use of intraoperative ultrasound is a relatively recent development and its first reported use in hepatobiliary surgery was by a Japanese group in 1958 to detect cholelithiasis [1,2]. At that time only A-mode ultrasound, which provides one-dimensional information in the form of a line graph, was available. B-mode ultrasound, which produces the familiar real-time two-dimensional image, became available in the mid-1970's. Makuuchi et al. reported on what was likely the first intraoperative ultrasound examination of the liver and pancreas using B-mode in 1977 [2,3]. Since that time the utilization of intraoperative ultrasound in hepatobiliary surgery has slowly increased as the capabilities of the available ultrasound machines have improved, most importantly the addition of color doppler imaging in the 1980's and the steady improvement in imaging resolution. Author CWH had the distinction of learning directly from Junji Machi, Paul Hansen, and Ellen Hagopian during residency, fellowship, and post graduate courses respectively. The techniques described here are the result of their teaching and adapted from the book *Abdominal Ultrasound for Surgeons* (Hagopian & Machi (eds.), 2014), specifically the chapter “Intraoperative and Laparoscopic Ultrasound During Liver Surgery” by Pittau, et al. [4].

## Indications

Intraoperative ultrasound in liver surgery is an important adjunct to preoperative imaging. It allows for thorough intraoperative evaluation of the liver in real-time. The dynamic nature of ultrasound enables imaging of vascular and biliary structures, segmental anatomy, and allows for recognition of any aberrant anatomy. Intraoperative ultrasound is sensitive for localizing liver tumors, both known and occult, which may have been missed on preoperative imaging. Thus, ultrasound enables precise intraoperative assessment of tumor resectability, planning of resection planes, identification of any clinically significant abnormalities and accurate tumor targeting during liver ablation. Ultrasound can also be useful post-resection or post-transplantation to confirm presence of adequate venous and arterial flow in the liver. However, it cannot be overstated enough that intraoperative ultrasound, whether laparoscopic or open, is a learned skill set which requires practice, time, and experience.

## Current evidence

A frequently asked question is whether intraoperative ultrasound is necessary given the high sensitivity and specificity of modern cross-sectional imaging. Studies attempting to address this question have focused mainly on comparing the sensitivity and specificity of ultrasound to MRI, and determining the rate at which intraoperative ultrasound changes clinical management. Focusing on literature from the last 5 years, current evidence supports the use of intraoperative ultrasound. Russolillo et al. [5] reported on 146 patients with colorectal liver metastases and found intraoperative ultrasound to be more sensitive than MRI (93.1 % versus 85.6 %) with similar specificity (96.5 % versus 98.6 %), and ultrasound findings altered the surgical plan in 13 % of cases. A similar 2019 study [6] of 721 patients with colorectal liver metastases also found a higher sensitivity for intraoperative ultrasound (94.5 %) when compared to MRI (75.1 %), with similar specificity (95.7 % versus 95.9 %). The surgical plan changed with the additional information provided by intraoperative ultrasound in 24 %. A 2018 study by Botea et al. [7] also echoed the importance of intraoperative ultrasound in surgical decision making; in 186 patients who underwent liver resection for various indications, the surgical plan was changed in 42 %. Similar results were seen in 175 patients with intrahepatic cholangiocarcinoma in whom intraoperative ultrasound altered the operation in 21 % of patients [8].

## Technique

### Equipment

**Fig. 1:**
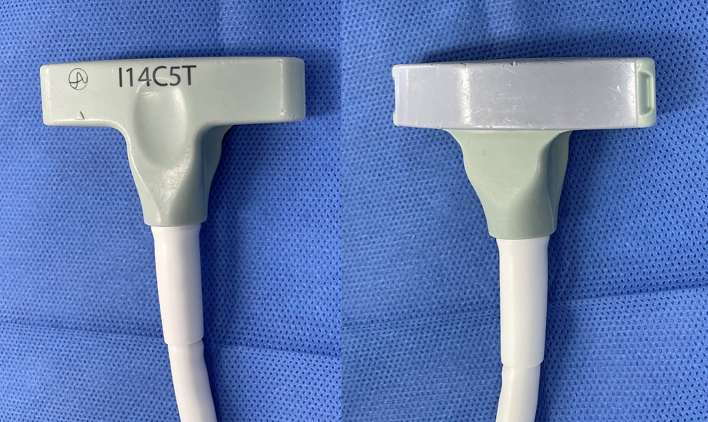
T-style probe for open intraoperative ultrasound.

**Fig. 2:**
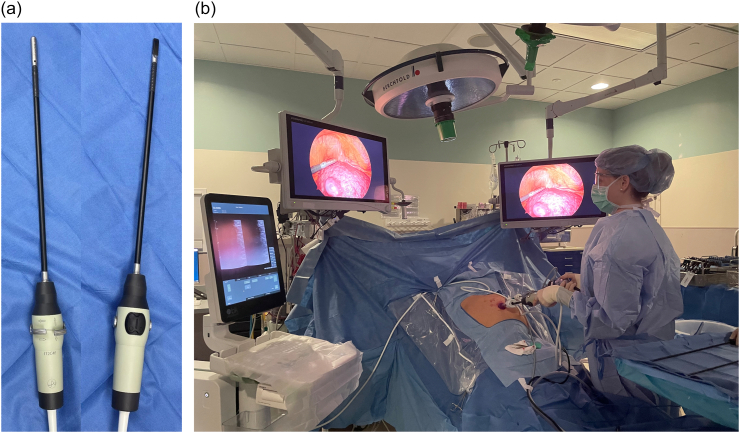
a) Flexible linear probe for laparoscopic intraoperative ultrasound, b) Typical operating room setup for performing laparoscopic liver ultrasound.

**Fig. 3:**
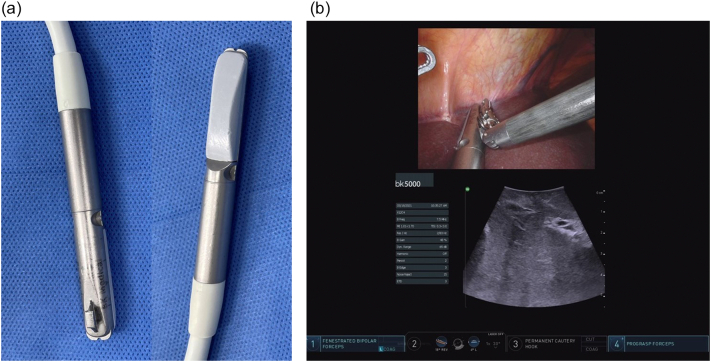
a) Curvilinear I-style probe for robotic intraoperative ultrasound, b) Surgeon's view on the robotic console enabling simultaneous visualization of both the robotic camera and ultrasound image.

Intraoperative ultrasound of the liver is ideally performed with dedicated multi-frequency transducers (5, 7.5 and 10 MHz). A higher frequency probe gives better image definition but will not image deeper structures, while a lower frequency probe will image deeper structures but at the cost of decreased image definition. When viewing the superficial liver, our preference is to use higher frequencies such as 10 MHz. Lower frequencies from 5 to 7.5 MHz are best used for a large, deep liver or one which is either steatotic or cirrhotic. All probes should have color Doppler capability. Typically, probes are sterile and do not require a probe cover. There are multiple types of open ultrasound probes, including T-probe linear or curvilinear arrays, T-style finger-grip, and I-style finger-grip (Fig. 1). Ultimately, the choice of probe is at the surgeon's discretion. The probe should fit comfortably in the palm of the surgeon's hand between the fingers to more easily explore the superior and right lateral segments of the liver. Laparoscopic probes are typically flexible linear or curvilinear arrays (Fig. 2a). A flexible probe allows for better contact with the liver surface, especially at the very superficial or right lateral segments. Current laparoscopic probes require a 10 mm port for insertion. In a typical operating room setup, the ultrasound machine and monitor screen are both placed on the patient's right side at or above the patient's arm level. This enables the surgeon to see both the ultrasound and laparoscopic displays simultaneously. A picture-in-picture display is also possible, where the laparoscopic image is displayed in one corner overlying the ultrasound image on a larger monitor screen. The surgeon stands on the patient's left side, opposite the machine (Fig. 2b). Robotic probes are typically curvilinear I-style arrays and also require a 10 mm port for insertion (Fig. 3a). Depending on the robotic machine, it may be possible to display the ultrasound image within the surgeon's visual platform at the console (Fig. 3b).

### Laparoscopic port placement

**Fig. 4:**
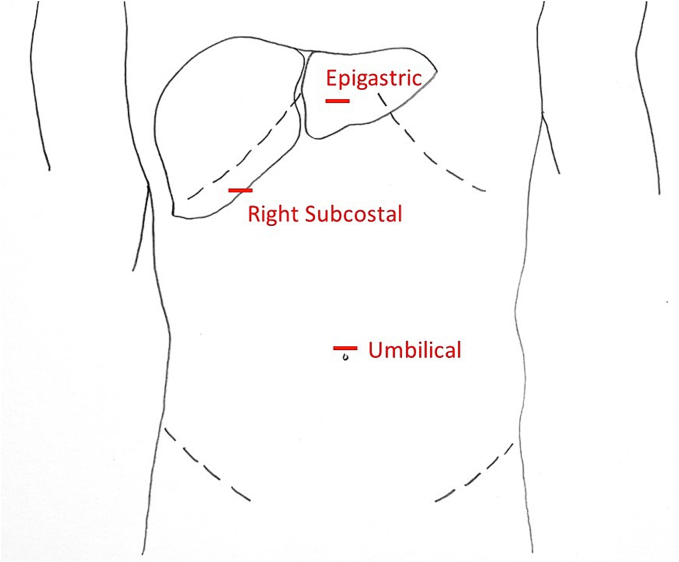
Port placement for laparoscopic intraoperative ultrasound of the liver.

As stated before, the laparoscopic ultrasound requires a 10 mm or larger port for insertion. Typically, epigastric or umbilical ports are useful for longitudinal imaging and lateral ports (i.e. subcostal position) are useful for transverse imaging (Fig. 4). For lesions located in the superior liver or at the dome, our preference is to place the ultrasound through a port in the epigastric region. For lesions in the left liver, we feel an umbilical port enables better visualization. Finally, we frequently use an umbilical port to evaluate the right liver, however if this does not provide adequate visualization we will switch the ultrasound to a right-sided subcostal port.

### Scanning technique

**Fig. 5:**
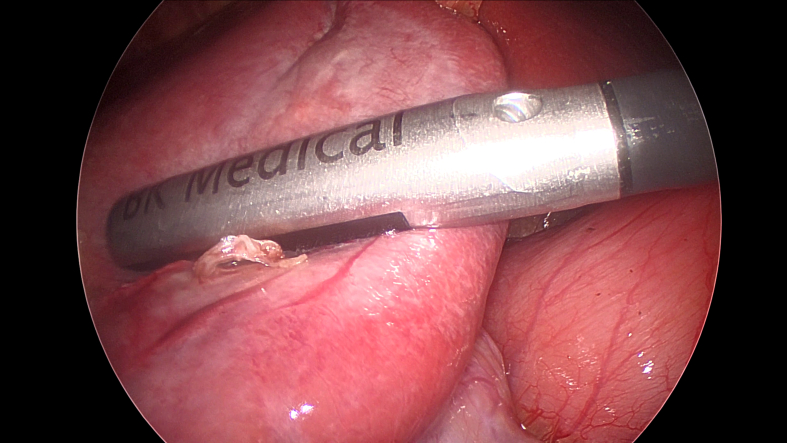
Contact (direct) scanning technique during liver ultrasound. Good probe contact with the liver surface is essential for optimal imaging.

**Fig. 6:**
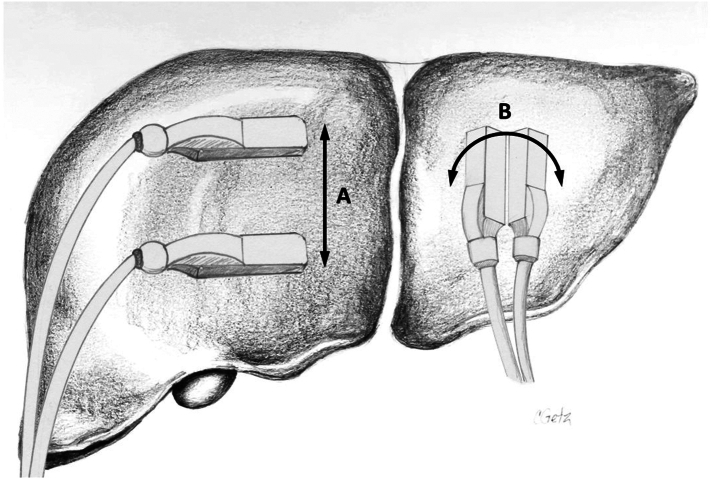
Liver scanning techniques involve (A) sliding and (B) rolling/tilting the ultrasound probe.

**Fig. 7:**
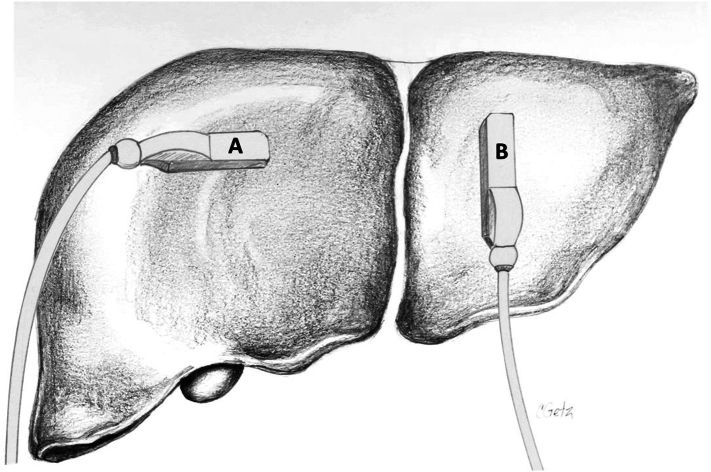
To ensure full visualization of all vascular and biliary structures as well as a complete parenchymal examination, the liver is viewed in two planes: (A) transverse and (B) longitudinal.

In general, direct contact scanning (Fig. 5) is used for intraoperative ultrasound of the liver. Although it is rarely needed, gel or saline can be used to improve the acoustic coupling. Techniques such as sliding and rolling (also referred to as tilting) the ultrasound probe allow complete visualization of the organ (Fig. 6). The liver is imaged in both the transverse (or sagittal) and longitudinal (or axial) planes (Fig. 7). Avoid heavy pressure on the liver with the ultrasound probe as this can deform the anatomy and compress the vascular structures, especially the thin-walled hepatic veins, making them difficult to identify. A standardized approach and technique are essential in order to ensure complete exploration of the liver. As mentioned previously, our approach, as presented here, is adapted from Pittau et al. [4] The technique of laparoscopic intraoperative ultrasound is similar to the open approach, although it relies more heavily on longitudinal views.

### Identification of the inferior vena cava and hepatic veins

**Fig. 8:**
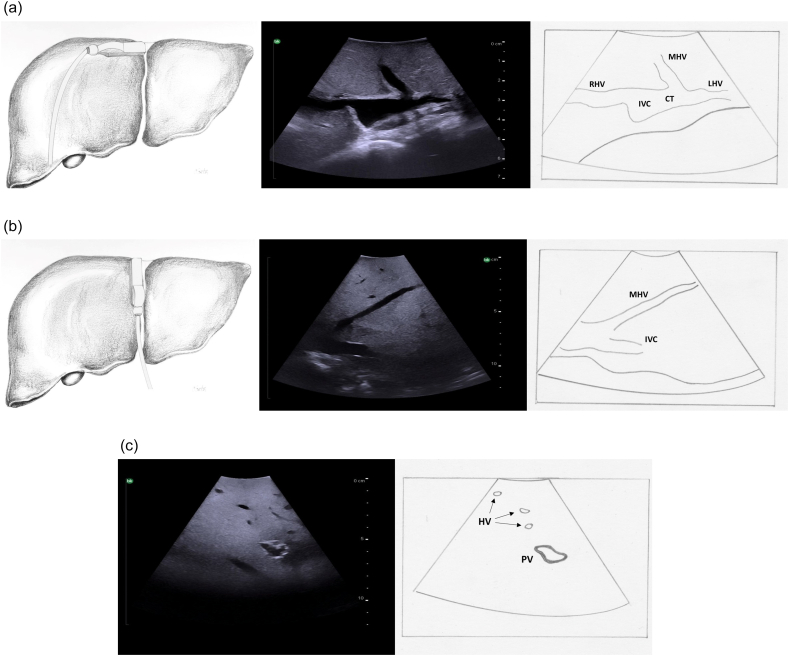
Intraoperative ultrasound of the origin of the hepatic veins as they join the inferior vena cava. a) Transverse probe placement and ultrasound image, b) Longitudinal probe placement and ultrasound image, c) Hepatic veins are recognized by thin echogenic walls in comparison to portal veins which have thick echogenic walls. RHV right hepatic vein, MHV middle hepatic vein, LHV left hepatic vein, CT common trunk, IVC inferior vena cava, HV hepatic veins, PV portal vein.

**Fig. 9:**
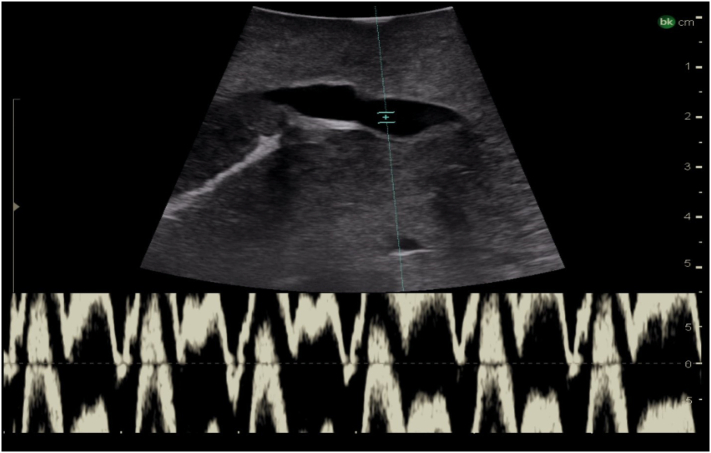
Intraoperative Doppler ultrasound of hepatic venous flow.

The initial step of intraoperative ultrasound of the liver is to identify each hepatic vein as it arises from the inferior vena cava. The probe is placed just to the right of the falciform ligament at the most superior border of the liver and is tilted up and angled slightly towards the heart (Fig. 8a, left panel). The hepatic veins are identified beginning at their junctions with the inferior vena cava. On transverse orientation a typical “rabbit ears” ultrasound image is obtained whereby the inferior vena cava appears as a circle (the rabbit's head) and the hepatic veins appear as the rabbit's ears (Fig. 8a, middle and right panel). On longitudinal orientation the hepatic veins are visualized as long tubes (Fig. 8b). In general, especially for larger vessels, the hepatic veins can be distinguished from the portal veins by the lack of a significant echogenic wall. (Fig. 8c). On color Doppler, hepatic veins demonstrate cyclical variations of flow velocity during cardiac pulsations with flow velocity reversal after contraction of the heart. They have predominantly antegrade flow (Fig. 9).

**Fig. 10:**
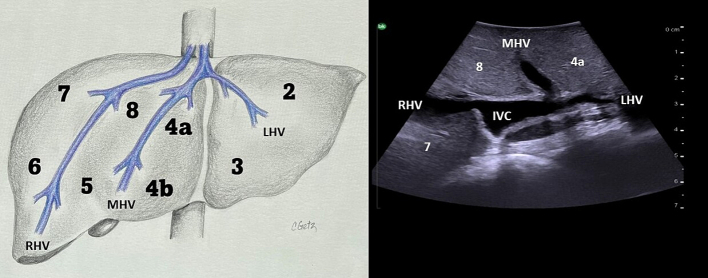
Anatomy of the hepatic veins in relation to the liver segments. RHV right hepatic vein, MHV middle hepatic vein, LHV left hepatic vein, IVC interior vena cava.

**Fig. 11:**
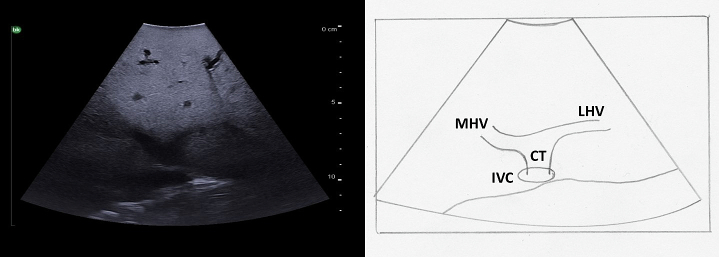
A common trunk is formed by the union of the left and middle hepatic veins before they empty into the inferior vena cava. MHV middle hepatic vein, LHV left hepatic vein, IVC inferior vena cava, CT common trunk.

**Fig. 12:**
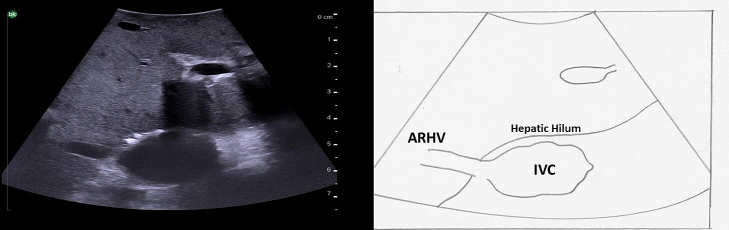
Accessory inferior right hepatic vein at the level of the hepatic hilum. IVC inferior vena cava, ARHV accessory inferior right hepatic vein.

**Fig. 13:**
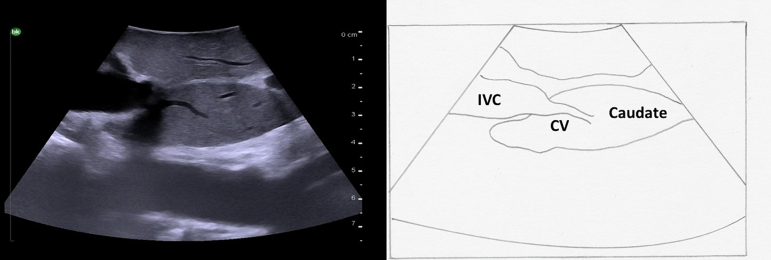
Small hepatic veins drain the caudate lobe directly into the inferior vena cava. Caudate, IVC inferior vena cava, CV caudate vein.

The hepatic veins run superiorly to inferiorly and can be useful in identifying the boundaries between the liver sections. (Fig. 10). The right hepatic vein separates the right anterior section (segments 5 and 8) from the right posterior section (segments 6 and 7). The middle hepatic vein separates the left and right hemiliver, specifically marking the boundary between the left medial section (segments 4a and 4b) and the right anterior section. The left hepatic vein identifies the boundary between segment 2 and segments 3/4. It is important to note, that despite what might be expected based on the anatomy of the right and middle hepatic veins, the left vein does not mark the boundary between the left medial section and the left lateral section (segments 2 and 3). In approximately 80 % of patients the middle hepatic vein and left hepatic vein merge to form a common trunk before emptying into the inferior vena cava (Fig. 8a, Fig. 11) The majority of patients (∼ 90 %) have a single right hepatic vein, however around one third also have an accessory inferior right hepatic vein (Fig. 12) [9]. It is important to identify these accessory hepatic veins as they have the potential for clinically significant bleeding during liver resections. Additionally, if present they can occasionally allow for the preservation of liver parenchyma in the inferior right liver (segments 5 and 6) even if the right hepatic vein is ligated. The caudate lobe is generally drained by 3 or 4 hepatic veins that are small, hard to visualize on ultrasound, and drain directly into the inferior vena cava (Fig. 13).

### Identification of the main portal vein, its bifurcation and pedicles

**Fig. 14:**
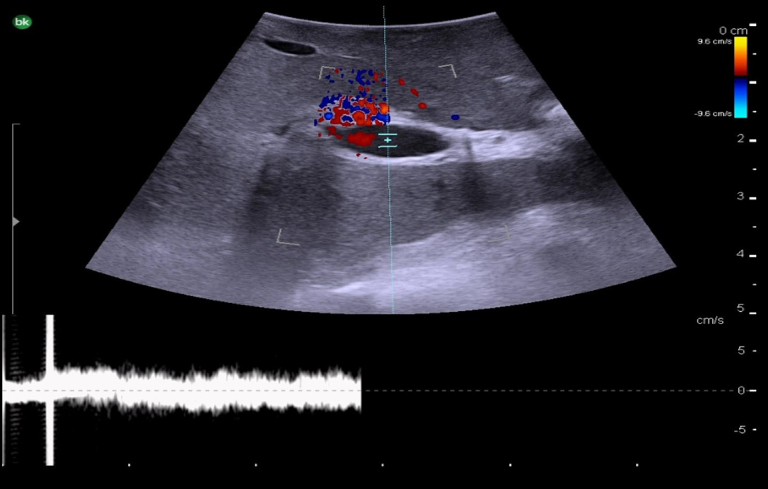
Intraoperative Doppler ultrasound of portal venous flow.

The next step is to identify the main portal vein, and then to follow each portal vein branch to its pedicles to define segmental anatomy. Portal veins are invested by Glisson's capsule and appear as thick-walled with a hyperechoic rim on ultrasound (Fig. 8c). On color Doppler, they have continuous antegrade flow towards the liver with variations in flow related to breathing (Fig. 14).

**Fig. 15:**
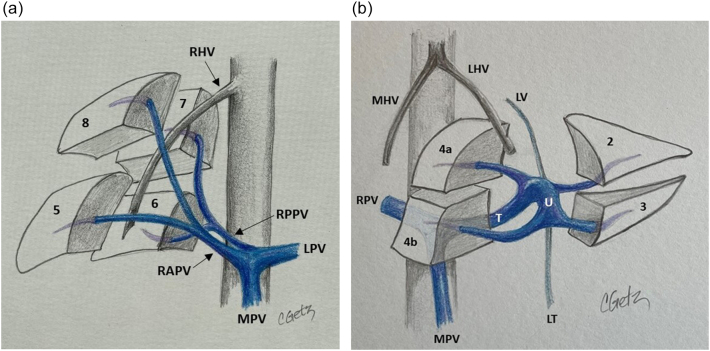
Portal venous anatomy of a) right portal vein and b) left portal vein. MPV main portal vein, RPV right portal vein, LPV left portal vein, RAPV right anterior portal vein, RPPV right posterior portal vein, T transverse portion of left portal vein, U umbilical portion of left portal vein, RHV right hepatic vein, MHV middle hepatic vein, LHV left hepatic vein, LT ligamentum teres, LV ligamentum venosum.

The main portal vein originates from the confluence of the splenic vein and the superior mesenteric vein. It then runs superiorly and to the patient's right. In the hilum of the liver, it divides into the right and left portal veins. The right portal vein runs horizontally to the patient's right and divides into anterior and posterior branches (Fig. 15a). The anterior branch divides into the anterosuperior branch which supplies segment 8 and the anteroinferior branch which supplies segment 5. The posterior branch divides into the posterosuperior branch to segment 7 and the posteroinferior branch to segment 6. The left portal vein is unique in liver anatomy, likely due to its role in fetal circulation. It is made up of two portions, the transverse and umbilical portion (Fig. 15b), and sits on the surface of the liver, unlike the right portal vein which is mostly intrahepatic. After the portal vein divides, the left portal vein continues to run superiorly and slightly to the patient's left (transverse portion). The umbilical portion of the left portal vein then travels from superior to inferior and in the fetal circulation provided a straight conduit for placental blood to travel from the umbilical vein to the ductus venosus on its way to the heart. After birth the umbilical vein becomes the ligamentum teres (round ligament) and the ductus venosus becomes the ligamentum venosum. The left portal vein gives off the segment 2 and 3 branches to the patient's left. The segment 2 branch comes off at the proximal (posterior) end of the umbilical portion and segment 3 branch comes off at the distal (inferior) end prior to its termination in the ligamentum teres. The segment 4 branches come off the umbilical portion of the left portal vein to the patient's right and are more variable in there positioning. Caudate branches arise varyingly off the right portal vein or the transverse portion of the left portal vein.

**Fig. 16:**
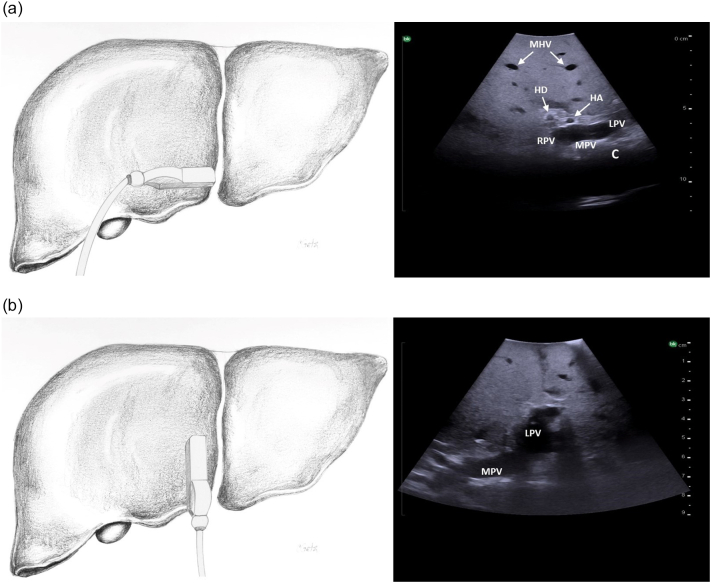
Intraoperative ultrasound of the portal vein bifurcation. a) Transverse probe placement and ultrasound image, b) Longitudinal probe placement and ultrasound image. MPV main portal vein, LPV left portal vein, RPV right portal vein, MHV middle hepatic vein tributaries, HD bile duct, HA hepatic artery, C caudate.

**Fig. 17:**
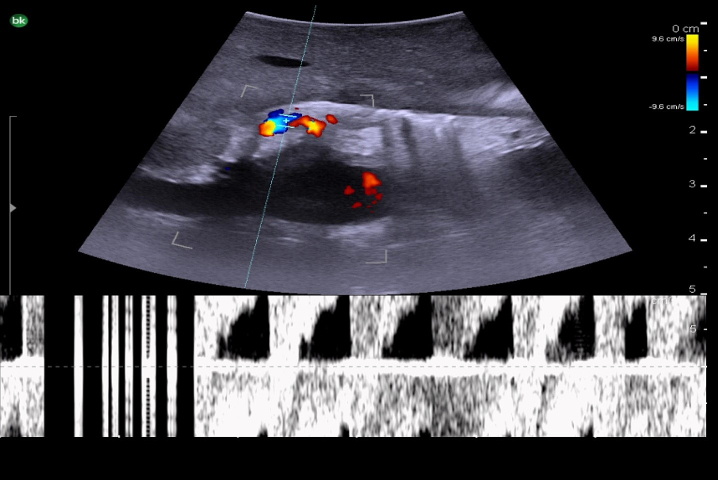
Intraoperative Doppler ultrasound of hepatic arterial flow. Note that there is no flow in the adjacent bile duct.

The probe is first placed transversely at the inferior border of the liver to the right of the falciform ligament overlying segment 4B with a slight angle towards the porta hepatis (Fig. 16a). If the portal vein bifurcation is not initially identified, it may be necessary to tilt the ultrasound probe towards the patient's feet, thereby sweeping the image plane inferiorly. When viewing the portal vein bifurcation on ultrasound the hepatic arteries and ducts can be seen immediately superficial to the bifurcation, with the middle hepatic vein more superficial. The arteries and ducts can be differentiated by color flow on Doppler ultrasound (Fig. 17). The caudate lobe can be seen deep to the bifurcation and deep to the caudate lobe is the inferior vena cava.

**Fig. 18:**
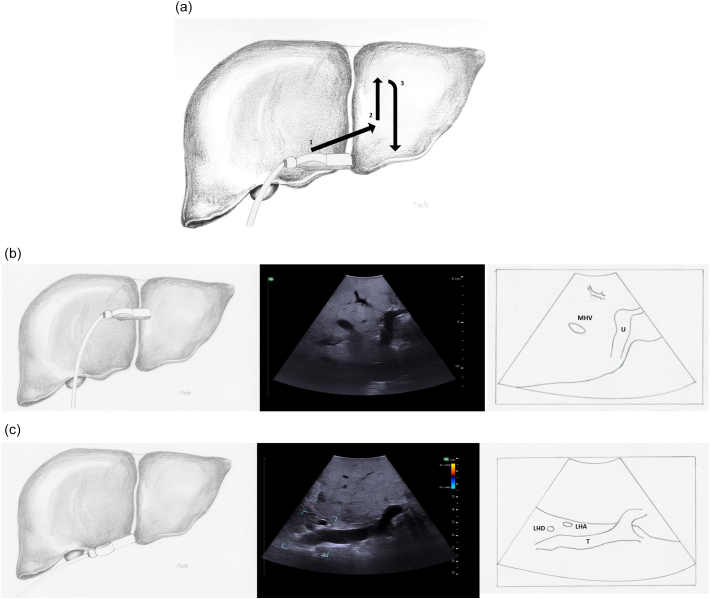
Intraoperative ultrasound of the left portal vein. a) The arrows represent the direction in which the ultrasound probe should be moved, while kept in a transverse orientation, to visualize the segmental branches of the left portal vein, b) Umbilical portion of the left portal vein, c) Transverse portion of the left portal vein. LHD left hepatic duct, LHA left hepatic artery, MHV middle hepatic vein, U umbilical portion of left portal vein, T transverse portion of left portal vein.

**Fig. 19:**
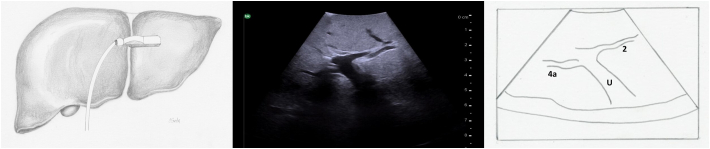
At the most superior portion of the left portal vein, the branch to segment 2 is seen to the patient's left and the branch to segment 4a is seen opposite (to the patient's right). U umbilical portion of left portal vein.

**Fig. 20:**
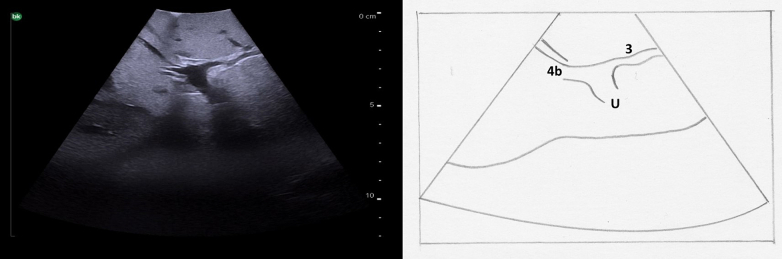
At the termination of the left portal vein, the branch to segment 3 is seen to the patient's left and the branch to segment 4b is seen opposite (to the patient's right). U umbilical portion of left portal vein.

Next, both the left and right portal veins are followed to their segmental branches. Beginning with the left portal vein, the ultrasound probe in a transverse orientation is moved, from its position over the bifurcation, superiorly and slightly to the patient's left along the transverse portion of the left portal vein (Fig. 18a arrow 1). Small caudate branches may be seen coming posteriorly off of the transverse portion of the left portal vein. Next the left portal vein should be followed superiorly along its umbilical portion until its most superior point (Fig. 18a arrow 2), where the segment 2 branch is usually seen coming off to the patient's left side and a branch to segment 4a may be seen coming off to the patient's right side (Fig. 19). The ultrasound probe is then moved inferiorly to follow the umbilical portion of the left portal vein distally (Fig. 18a arrow 3). As the vein tracks inferiorly it tends to course more superficially. Again, the portal venous supply to segment 4 is more variable but can generally be identified as branches coming off to the patient's right. The left portal vein terminates at the ligamentum teres or round ligament, which is seen as a hyperechoic zone on ultrasound. At the termination of the left portal vein, the branch to segment 3 can usually be seen coming off to the patient's left side (Fig. 20).

**Fig. 21:**
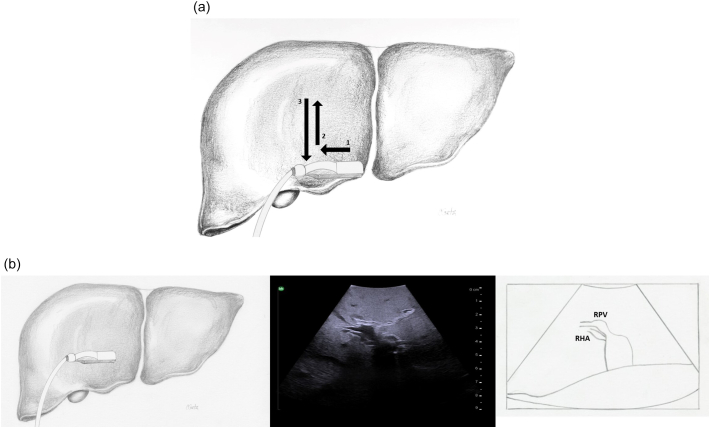
Intraoperative ultrasound of the right portal vein. a) The arrows represent the direction in which the ultrasound probe should be moved, while kept in a transverse orientation, to visualize the segmental branches of the right portal vein b) Transverse probe placement ultrasound image. RPV right portal vein, RHA right hepatic artery.

**Fig. 22:**
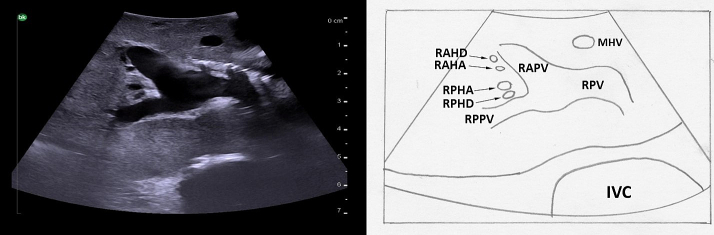
The right portal vein is short and bifurcates early into the right anterior and posterior branches. Biliary obstruction in this case allows for visualization of the dilated hepatic ducts. RPV right portal vein, RAPV right anterior portal vein, RPPV right posterior portal vein, RAHD dilated right anterior hepatic duct, RAHA right anterior hepatic artery, RPHA right posterior hepatic artery, RPHD dilated right posterior hepatic duct, MHV middle hepatic vein, IVC inferior vena cava.

To follow the right portal vein, the ultrasound probe is placed to the right of the falciform ligament, continuing to use a transverse orientation, and moved towards the patient's right (Fig. 21a arrow 1). The right portal vein is much shorter, and has an early division into its anterior and posterior branches (Fig. 22). When the bifurcation is visualized, the right anterior portal vein is seen superficially and the right posterior portal vein is deep; both run from medial to lateral. Midway between the anterior and posterior branches, the right hepatic vein can be seen running from superior to inferior. Moving the ultrasound probe superiorly from this position will reveal the segment 8 branch superficially and the segment 7 branch deep (Fig. 21a arrow 2). Moving the ultrasound inferiorly will reveal the segment 5 branch superficially and the segment 6 branch deep (Fig. 21a arrow 3).

**Fig. 23:**
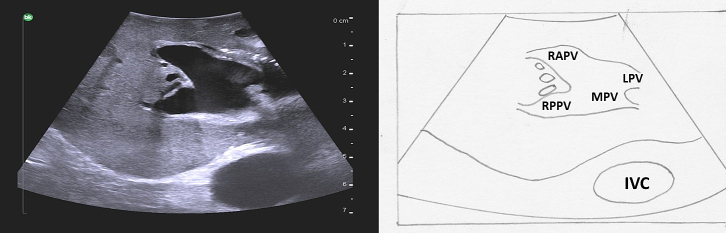
A portal vein trifurcation; the anterior and posterior right portal veins arise directly off the main portal vein at the same location as the left portal vein. There is no true right portal vein. MPV main portal vein, LPV left portal vein, RAPV right anterior portal vein, RPPV right posterior portal vein, IVC inferior vena cava.

Again, it is important to identify any anomalous anatomy. For example, a portal vein trifurcation in which there is no true right portal vein and the right anterior and posterior portal veins arise off of the main portal vein (Fig. 23) occurs in approximately 7 % of patients [10]. This is especially important to consider when performing either a right anterior or right posterior sectionectomy.

Identification of the Hepatic Arteries.

**Fig. 24:**
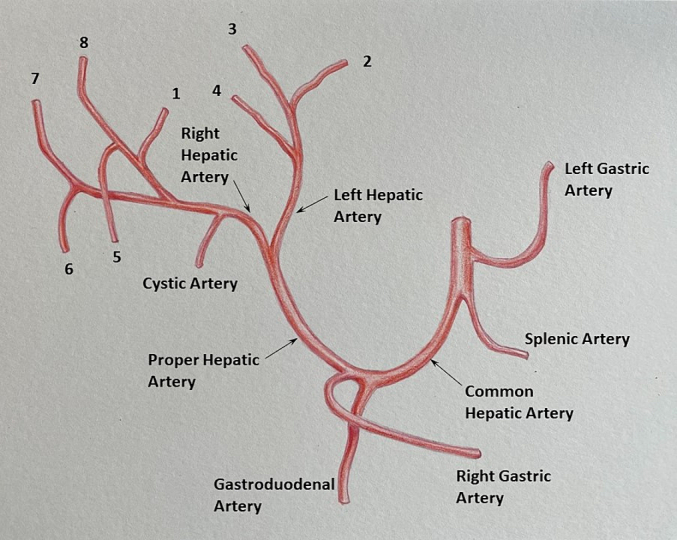
Conventional hepatic arterial anatomy. Adapted from Gray's Anatomy: the anatomical basis of clinical practice, 40th edition, Standring S (editor), 2008, Fig. 68.9A, with permission from Elsevier.

**Fig. 25:**
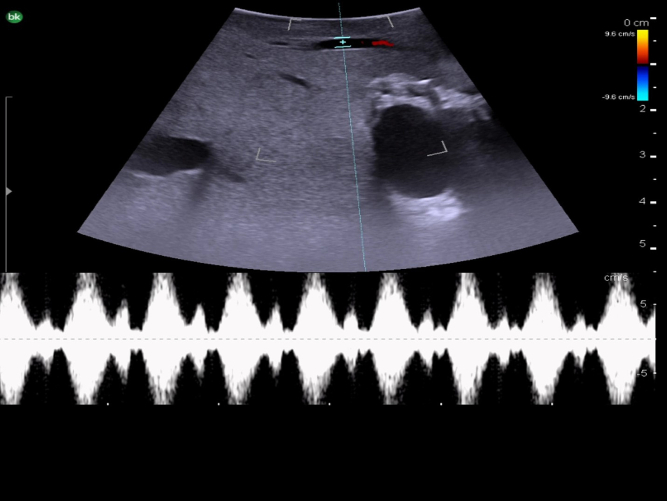
Intraoperative Doppler ultrasound of hepatic arterial flow.

Hepatic arterial anatomy, which defines the segmental anatomy of the liver, is also important to identify (Fig. 24). After its origin from the celiac trunk, the common hepatic artery runs anteriorly and to the patient's right before dividing into 3 branches (left gastric artery, common hepatic artery, and splenic artery). The hepatic artery then travels anteromedial to the portal vein, ascending within the hepatoduodenal ligament, and bifurcates in the porta hepatis into the right and left hepatic arteries. The left hepatic artery divides to form segment 2, 3, and 4 branches. The right hepatic artery typically passes anterior to the main portal vein, gives off the cystic artery, and subsequently bifurcates to form the right anterior (supplying segments 5 and 8) and right posterior (supplying segments 6 and 7) branches. On color Doppler, hepatic arteries have a low resistance waveform with brisk systolic uptake and continuous antegrade flow during diastole (Fig. 25).

**Fig. 26:**
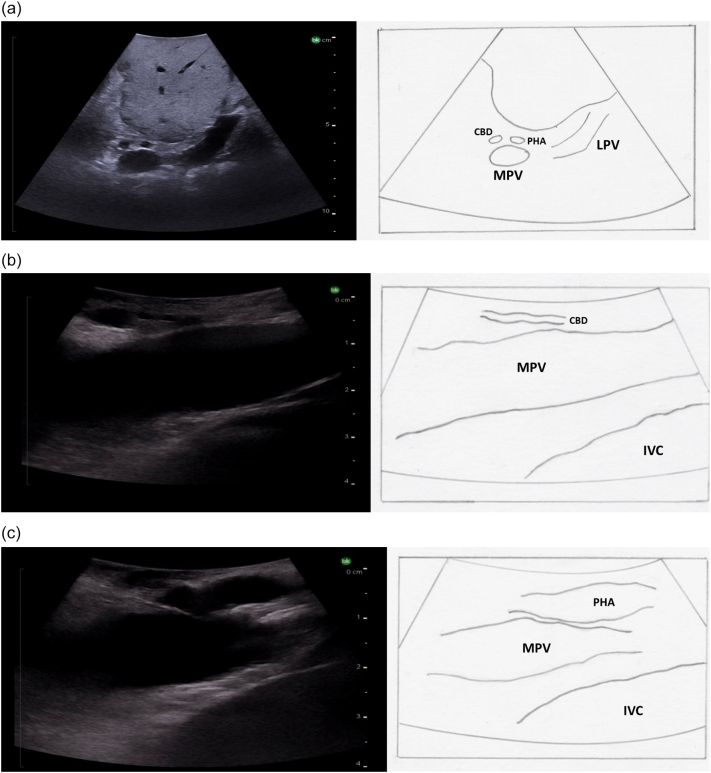
Intraoperative ultrasound of the hepatoduodenal ligament. Ultrasound image with the probe in the transverse orientation (a). Placing the probe longitudinally over the lateral aspect of the hepatoduodenal ligament visualizes the bile duct (b) and sliding the probe medially will visualize the proper hepatic artery. MPV main portal vein, PHA proper hepatic artery, CBD common bile duct, LPV left portal vein, IVC inferior vena cava.

**Fig. 27:**
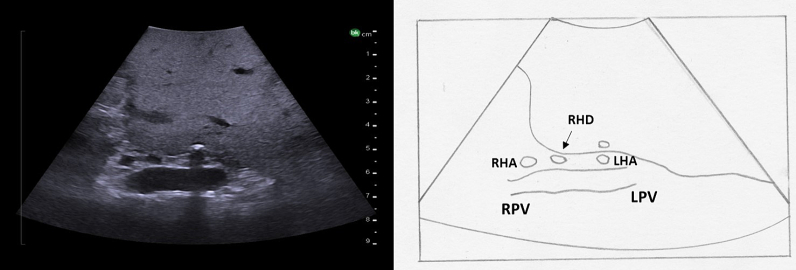
Ultrasound image demonstrating the bifurcation of the proper hepatic artery into the left and right hepatic arteries. RPV right portal vein, LPV left portal vein, RHA right hepatic artery, RHD right hepatic duct, LHA left hepatic artery.

Starting with the ultrasound probe in a transverse orientation over the hepatoduodenal ligament (Fig. 26a), the “Mickey Mouse” view can be visualized with the main portal vein posteriorly, the proper hepatic artery anteriorly and to the patient's left, and the common bile duct anteriorly and to the patient's right. The ultrasound probe is then moved superiorly on the hepatoduodenal ligament to visualize the hepatic artery bifurcation (Fig. 27). Hepatic arteries run with portal veins and bile ducts within the pedicles, and can be differentiated from either of these two structures by Doppler flow (Fig. 17). Intrahepatic arteries are not usually visible as they are very small.

Important anatomic variations include a replaced right hepatic artery off the superior mesenteric artery, occurring in approximately 10 % of patients. This can be seen traveling posterior to the portal vein. A replaced left hepatic artery off of the left gastric artery, occurring in approximately 16 % of patients, passes posterior to the left lateral segments and then runs through the ligamentum venosum [11].

### Identification of the bile duct and its main branches

**Fig. 28:**
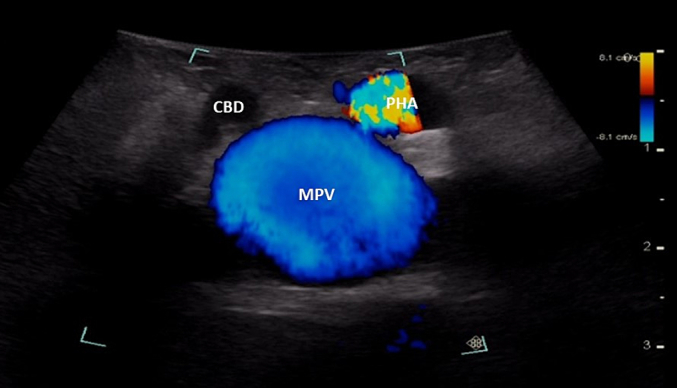
A lack of blood flow on Doppler ultrasound differentiates the bile duct from the hepatic artery and portal vein. MPV main portal vein, CBD common bile duct, PHA proper hepatic artery.

**Fig. 29:**
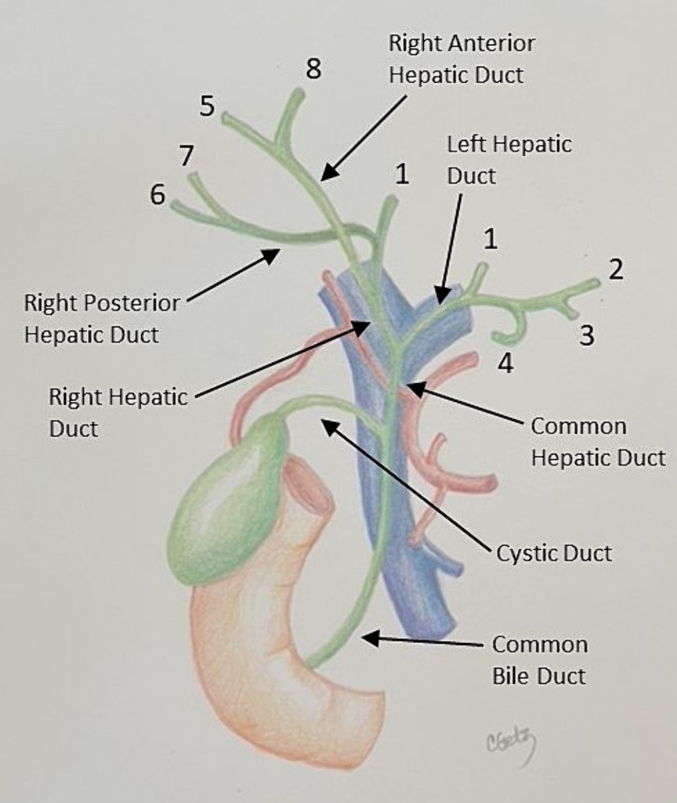
Anatomy of the biliary tree. Note that the common bile duct is anteromedial to the portal vein and that when conventional hepatic arterial anatomy is present, the right hepatic artery runs posterior to the common hepatic duct, and passes laterally and posterior to the right hepatic duct. Adapted from Surgery, 38(8), Mahadevan V, Anatomy of the gallbladder and bile ducts, Fig. 1, with permission from Elsevier.

The last anatomic structures to identify are the hepatic ducts. The common bile duct runs in the hepatoduodenal ligament anteromedial to the portal vein. Again, a lack of flow on color Doppler can be used to distinguish the bile duct from the vascular structures (Fig. 28). The common bile duct transitions to the common hepatic duct where the cystic duct enters and bifurcates into the right and left hepatic ducts at the porta hepatis. The hepatic ducts then follow the hepatic arteries to give off segmental bile ducts (Fig. 29).

**Fig. 30:**
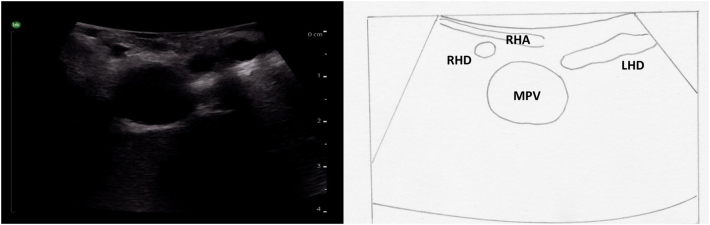
The bifurcation of the left and right hepatic ducts is seen in the superior portion of the hepatoduodenal ligament on intraoperative ultrasound. MPV main portal vein, RHD right hepatic duct, LHD left hepatic duct, RHA right hepatic artery.

The ultrasound probe is again placed transversely on the hepatoduodenal ligament to obtain the “Mickey Mouse” view (Fig. 26a, Fig. 28). The ultrasound probe is moved superiorly up the hepatoduodenal ligament to visualize the bifurcation of the right and left hepatic ducts (Fig. 30). More peripheral bile ducts are not usually seen as they are very small. They may be visualized, however, in the setting of biliary obstruction when they become dilated (Fig. 22).

One important anatomic variation to recognize is the anomalous insertion of a right posterior sectoral hepatic duct into the left hepatic duct or directly into the main hepatic duct inferior to the hepatic hilum.

### Systematic parenchymal scan

**Fig. 31:**
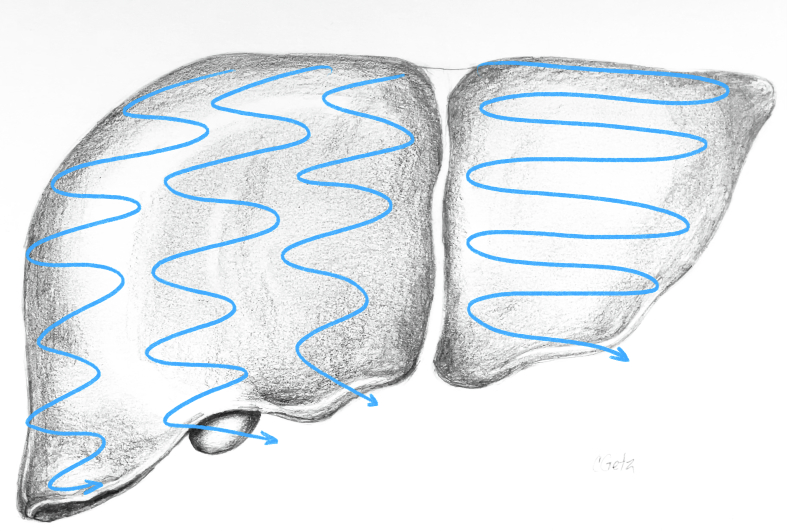
The lawnmower technique is one method to ensure systematic and complete parenchymal scanning. The ultrasound probe is moved from left to right in small vertical stripes staring at the superior liver and moving inferiorly within each liver section.

**Fig. 32:**
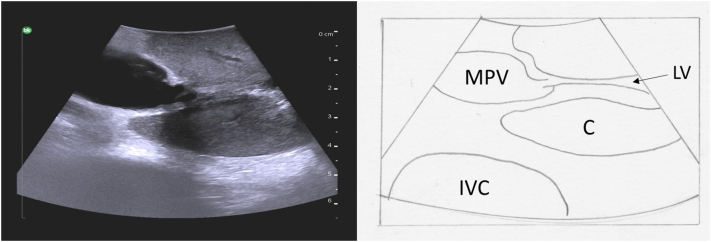
Intraoperative ultrasound with a transverse view of the caudate lobe. MPV main portal vein, IVC inferior vena cava, C caudate, LV ligamentum venosum.

The final step in performing a complete liver ultrasound is a systematic parenchymal scan. Again, this is performed in 2 different planes starting with transverse and then longitudinal, ensuring that all areas are explored. It is important to ensure that whichever technique is used, consistency is achieved across all scans to ensure that no area of the liver is missed. One example is the lawnmower technique, back and forth sweeping movements within each section (Fig. 31). The caudate lobe can be visualized by placing the ultrasound probe in transverse orientation over the left medial liver (Fig. 32). It is seen deep to the portal vein bifurcation and superficial to the inferior vena cava, as mentioned previously.

**Fig. 33:**
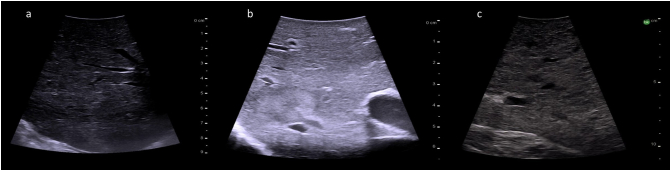
Intraoperative ultrasound images of liver parenchyma. a) Normal liver parenchyma (Frequency 10 MHz), b) Hepatic steatosis (Frequency 10 MHz), c) Cirrhosis (Frequency 5 MHz).

The normal liver parenchyma is of a medium echogenicity and is made of many thin spots creating a homogenous appearance (Fig. 33a). In comparison to the kidney, the liver is less echogenic. Steatosis results in an increase in liver echogenicity, but with a smooth regular liver surface (Fig. 33b). A cirrhotic liver will have an irregular and nodular surface with increased liver echogenicity on ultrasound (Fig. 33c).

**Fig. 34:**
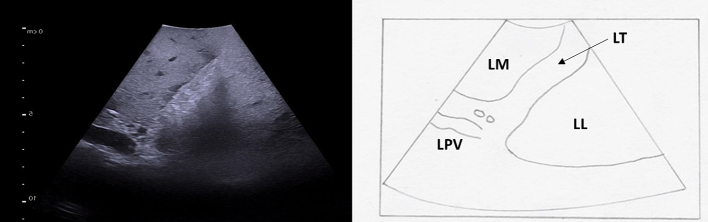
The ligamentum teres (round ligament) is seen as a hyperechoic zone at the free edge of the falciform ligament on intraoperative ultrasound. The left portal vein terminates here, giving off its branches to segment 3 and 4b. LT ligamentum teres, LM left medial, LL left lateral, LPV left portal vein.

**Fig. 35:**
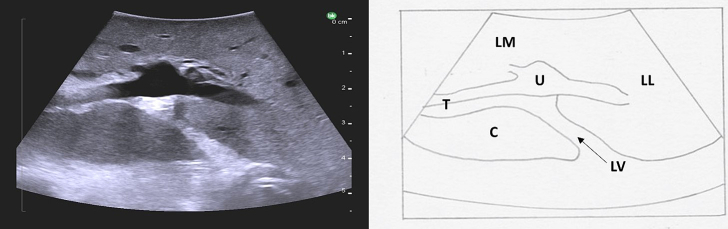
The ligamentum venosum is a hyperechoic zone separating the caudate lobe from the base of the left portal vein. LV ligamentum venosum, T transverse portion of the left portal vein, U umbilical portion of left portal vein, LM left medial liver, LL left lateral liver, C caudate.

Hepatic ligaments appear as hyperechoic structures. The round ligament, or ligamentum teres, can be visualized at the free edge of the falciform ligament (Fig. 34). The ligamentum venosum separates the caudate lobe from the left liver, and can be seen at the base of the left portal vein (Fig. 32, Fig. 35).

## Ultrasound features of hepatic tumors

Liver masses are best differentiated by their appearance on ultrasound. Tumors are characterized as being anechoic, hyperechoic, hypoechoic or isoechoic when compared to normal hepatic parenchyma.

**Fig. 36:**
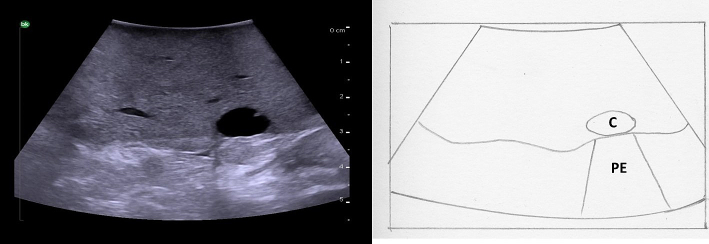
Intraoperative ultrasound appearance of an anechoic liver cyst with posterior enhancement. C cyst, PE posterior enhancement.

Anechoic masses are not echogenic and appear black on ultrasound. These are usually fluid-filled (cystic). Simple cysts are typically thin-walled without septations or nodularity. They demonstrate posterior enhancement as the ultrasound beam encounters cyst fluid which is weekly attenuating (Fig. 36). Abscesses can appear similarly, however, the internal fluid is typically complex. Biliary cystadenomas are typically thick-walled with multiple septations and may have nodularity.

**Fig. 37:**
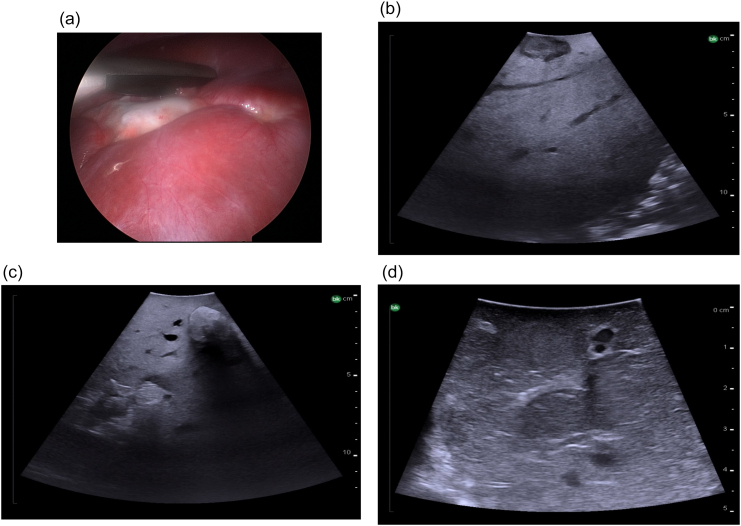
Intraoperative ultrasound appearance of colorectal liver metastases, a) Laparoscopic view of colorectal liver metastasis which is fibrotic, b) Hypoechoic metastasis with surrounding hypoechoic rim of fibrosis, c) Hyperechoic metastases, d) Isoechoic metastases.

Hypoechoic masses have an echogenicity that is less than the background liver. They are typically malignant. Most liver metastases (colorectal, neuroendocrine, melanoma, pancreatic cancer) appear this way on ultrasound. There is typically also a hypoechoic rim corresponding with fibrosis (Fig. 37b). Of note the appearance of colorectal liver metastases can be variable; they can also appear hyperechoic (Fig. 37c) or isoechoic (Fig. 37d).

**Fig. 38:**
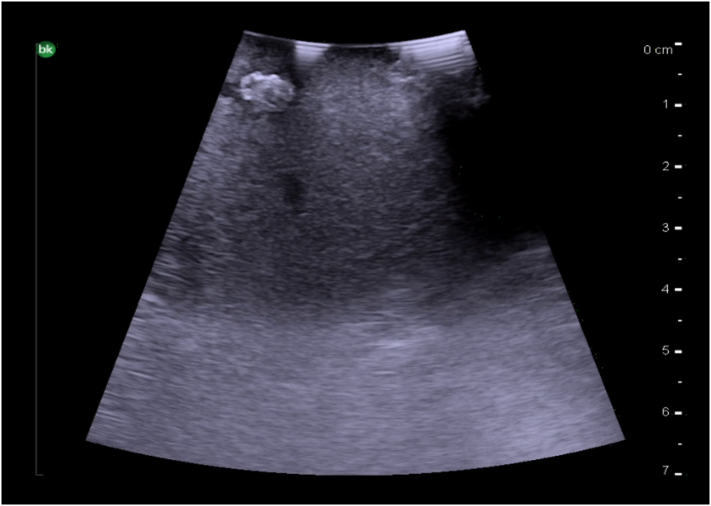
Intraoperative ultrasound appearance of a hemangioma (hyperechoic).

Hyperechoic liver masses are more echogenic than the background liver, and usually represent benign tumors. Hepatic adenomas are usually bright and well-circumscribed. Hemangiomas may appear similarly but with a more irregular border (Fig. 38).

**Fig. 39:**
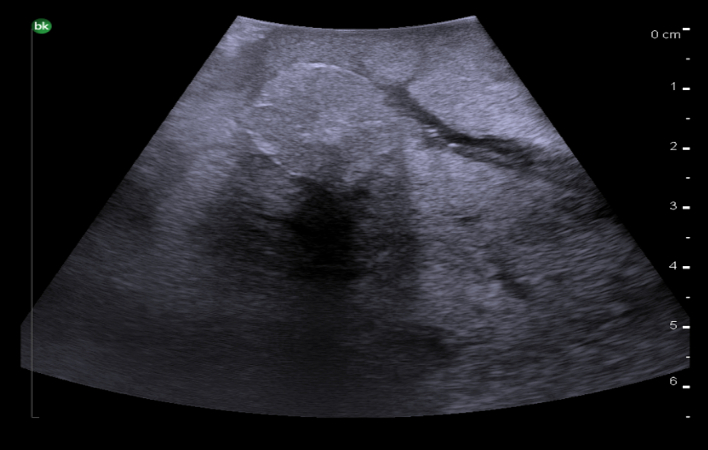
Intraoperative ultrasound appearance of an isoechoic hepatocellular carcinoma with a hypoechoic rim.

Isoechoic liver masses have an echogenicity similar to the background liver so can be difficult to identify. A hypoechoic rim or mass effect on adjacent vascular structures is typically present in order to identify these masses. Focal nodular hyperplasia is usually isoechoic, well-circumscribed and has a central feeding artery on Doppler ultrasound. Hepatocellular carcinoma can also be isoechoic and typically has a hypoechoic rim (Fig. 39).

## Troubleshooting

**Fig. 40:**
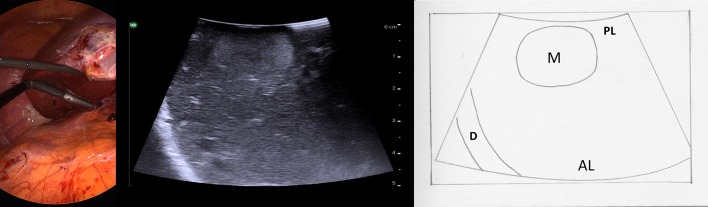
Posterior liver surface contact scanning with the laparoscopic and ultrasound view. M mass, D diaphragm, PL posterior liver, AL anterior liver.

Even in the most experienced hands there are many situations in which intraoperative ultrasound is challenging. Some areas of the liver can be particularly difficult to visualize, for example the superior, far lateral, and deep posterior aspect of the right liver, such as segment 7. To explore this “blind area,” medial displacement of the liver can be maximized by placing the patient in a left semi-lateral position with the right side elevated and by mobilizing the right triangular ligament. Adjunctive maneuvers also include using a standoff technique with saline immersion or contact scanning from the posterior liver surface (Fig. 40). It is easy to miss very superficial lesions within the first 5 mm of the surface of the liver due to decreased resolution of the ultrasound as well as compression by the probe. Often, either contact scanning from the posterior liver surface or a probe standoff technique can be useful there also.

**Fig. 41:**
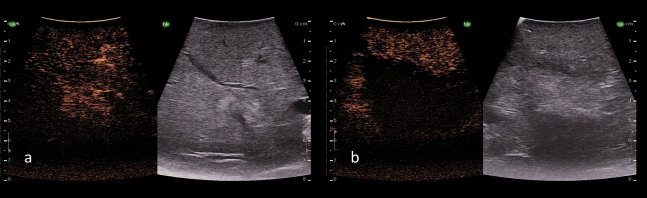
Intraoperative ultrasound of a tumor using a microbubble contrast agent, a) Before thermal ablation the tumor enhances on contrast ultrasound demonstrating viable cells with blood flow, b) After thermal ablation blood flow can no longer be appreciated in the tumor and it appears dark on contrast ultrasound.

Of particular note are hard to see lesions such as disappearing colorectal liver metastases, malignant nodules in the background of a cirrhotic liver, and areas of tumor recurrence within a post-ablation cavity. The importance of using tumor location in relation to intrahepatic vasculature cannot be understated. Newer ultrasound technologies use contrast software which enables more detailed information about tumor vascularity and tissue microcirculation to be obtained. Intravenous microbubble contrast agents can be used to enhance the detection of some of these difficult to find lesions (Fig. 41).

## Conclusion

Intraoperative ultrasound provides crucial diagnostic and staging information for the surgeon during liver surgery. The use of ultrasound should be considered mandatory during hepatic surgery and should be part of every surgeon's professional training and experience. Despite the high quality of preoperative imaging, intraoperative ultrasound is still an essential tool in detecting lesions and planning and executing the surgical strategy.

## Funding sources

None.

## Ethics approval

As per our institutional guidelines and applicable regulations, ethics approval was not necessary for this study as it did not involve human subjects, animal experimentation, or the use of identifiable patient information.

## CRediT authorship contribution statement

**Natasha Leigh:** Conceptualization, Writing – original draft. **Chet W. Hammill:** Conceptualization, Writing – original draft, Writing – review & editing.

## Declaration of competing interest

The authors hereby declare that there are no conflicts of interest with regards to the submission of the above-mentioned manuscript. We affirm that the research conducted and the content presented in the manuscript are free from any financial, personal, professional, or academic relationships or affiliations that could be perceived as having the potential to bias or influence the research or the manuscript's conclusions.
